# The role of preoperative aspartate aminotransferase-to-platelet ratio index in predicting complications following total hip arthroplasty

**DOI:** 10.1186/s12891-023-07063-9

**Published:** 2023-12-02

**Authors:** M. A. McLellan, M. R. Donnelly, K. T. Callan, B. E. Lung, S. Liu, R. DiGiovanni, W. C. McMaster, R. N. Stitzlein, S. Yang

**Affiliations:** 1 Department of Orthopaedic Surgery, University of California Irvine, 101 The City Drive South, Pavilion III, Building 29A, Orange, CA 92868 USA; 2 Department of Orthopaedic Surgery, New York University, New York, USA; 3 Stony Brook School of Medicine, New York, USA

**Keywords:** Total hip arthroplasty, Osteoarthritis, Liver damage, Cirrhosis, APRI, Complication

## Abstract

**Background:**

The purpose of this study was to investigate the relationship between preoperative aspartate aminotransferase-to-platelet ratio index (APRI) and postoperative complications following total hip arthroplasty (THA).

**Methods:**

All THA for osteoarthritis patients from 2007 to 2020 within the American College of Surgeons (ACS) National Surgical Quality Improvement Program (NSQIP) database were included in this study. Subjects were subsequently divided into cohorts based on APRI. Four groups, including normal range, some liver damage, significant fibrosis, and cirrhosis groups, were created. Comparisons between groups were made for demographics, past medical history, and rate of major and minor complications. Other outcomes included readmission, reoperation, discharge destination, mortality, periprosthetic fracture, and postoperative hip dislocation. Multivariate logistic regression analysis was performed to determine the role of preoperative APRI in predicting adverse outcomes. Statistical significance was set at *p* < 0.05.

**Results:**

In total, 104,633 primary THA patients were included in this study. Of these, 103,678 (99.1%) were in the normal APRI group, 444 (0.4%) had some liver damage, 256 (0.2%) had significant fibrosis, and 253 (0.2%) had cirrhosis. When controlling for demographics and relevant past medical history, the abnormal APRI groups had a significantly higher likelihood of major complication, minor complication, intraoperative or postoperative bleeding requiring transfusion, readmission, and non-home discharge (all *p* < 0.05) compared to normal APRI individuals.

**Conclusions:**

Abnormal preoperative APRI is linked with an increasing number of adverse outcomes following THA for osteoarthritis for patients across the United States.

**Level of evidence:**

Level I

## Introduction

Total hip arthroplasty (THA) is a highly successful procedure for relieving pain and lessening disability in individuals with end-stage degenerative joint disease [1–3]. By some estimates, there were around 2.5 million individuals living with a THA in the United States in the year 2010 [4]. Furthermore, some researchers have predicted that the number of annual THA procedures will nearly double by 2040 and increase by 659% by the year 2060 [3, 4]. Given, then, the rapid growth of and demand for THA in the United States, it is vital that efforts are made to optimize these patients in order to avoid adverse postoperative outcomes. This includes an in-depth perioperative risk assessment [4] and a conversation with patients about expected outcomes based on their comorbid conditions.

One comorbid condition that has been well-studied and widely reported on in the orthopaedic literature is cirrhosis. It has been found that for patients undergoing inpatient orthopaedic procedures, cirrhosis increases the risk for postoperative mortality, lengthens the hospital stay, and causes a spike in total costs [5–7]. With regards to cirrhosis in THA, specifically, a systematic review performed in 2019 determined that patients with liver disease experienced greater rates of infection, aseptic hardware loosening, and periprosthetic fracture [8] while other studies have shown additional intensive care unit requirements, mortality, unplanned hospital readmission, and postoperative bleeding requiring transfusion for these patients [8].

To combat these grave complications, some study authors have recommended preoperative assessment and medical optimization by a hepatologist [9, 10]. Amongst hepatologists, currently, the gold standard for diagnosis and monitoring liver fibrosis is with liver biopsy and histological examination [11]. However, in the past few years, multiple biochemical markers have been suggested as noninvasive alternatives for the evaluation of liver fibrosis and have been shown to be more cost effective [12]. For example, the aspartate aminotransferase (AST) to platelet ratio index (APRI) is a new, non-invasive measure to assess liver damage with laboratory testing alone. Already, it has been applied to and validated for surgical preoperative risk assessments in the vascular surgery and general surgery literature [13]. However, to our knowledge, APRI as a risk stratification tool has not yet been well-documented in the field of orthopedics. Thus, the purpose of this study was to investigate the relationship between preoperative APRI and postoperative complications following THA in the United States.

## Methods

**Fig. 1:**
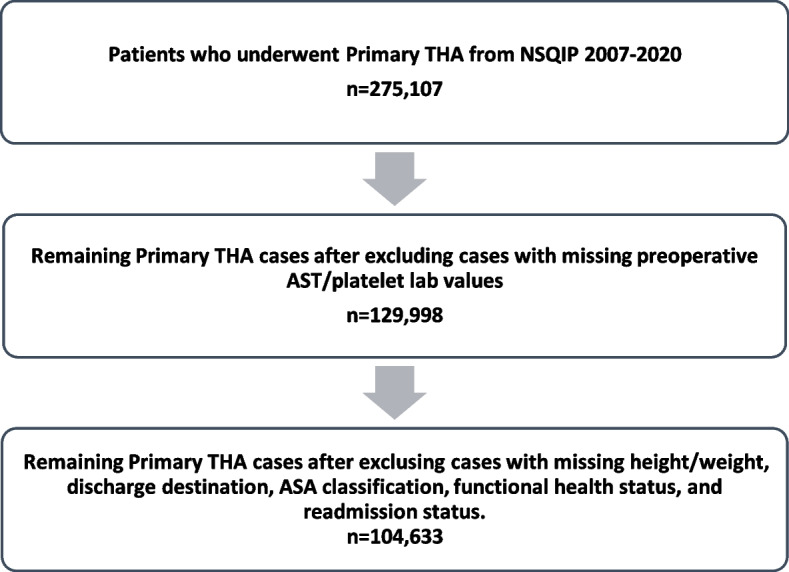
Case selection schematic. THA, total hip arthroplasty; NSQIP, National Surgical Quality Improvement Program; ASA, American Society of Anesthesiologists

This retrospective study utilized a national, de-identified, and publicly available database and, as such, no informed consent was obtained. The study was considered exempt by the Institutional Review Board and no funding was provided. The American College of Surgeons (ACS) National Surgical Quality Improvement Program database (NSQIP) was queried using Current Procedural Terminology (CPT) code “27,130” to identify patients who underwent primary THA in the United States in the years 2007 to 2020. Subjects were included if they were aged ≥ 18 years and if the indication for primary THA was osteoarthritis. Subjects were excluded if they were missing preoperative AST or platelet data, as these are used to calculate the APRI. Subjects were additionally excluded if they had the following information missing in the NSQIP database: height or weight, discharge destination, American Society of Anesthesiologists (ASA) class, or baseline functional status (Fig. 1).

Data collected from the NSQIP database for these patients included demographic data, such as sex and age, and past medical history information, such as body mass index (BMI), ASA class, smoking status, chronic steroid use, hypertension, congestive heart failure, diabetes mellitus, chronic obstructive pulmonary disease, and bleeding disorders. The 5-Factor Modified Frailty Index was also calculated as a summation of the presence of five past medical conditions: congestive heart failure, diabetes mellitus, chronic obstructive pulmonary disease or current pneumonia, hypertension requiring medication, and non-independent functional status [14]. All complication data was also extracted, and categories for major versus minor complications were created. “Major” complications were defined as deep incisional surgical site or organ/space infection, cardiac arrest, myocardial infarction, deep vein thrombosis, pulmonary embolism, stroke, unplanned reintubation, remaining on a ventilator for over 48 h, sepsis, septic shock, readmission, reoperation and/or 30-day mortality. “Minor” complications were superficial incisional surgical site infection, wound dehiscence, pneumonia, urinary tract infection and/or blood transfusion received within 72 h of the procedure.

For all patients, four cohorts based on APRI were created. Preoperative APRI was calculated using the equation ((measured AST/40) *100)/platelets [5]. The number 40 was used to represent the upper limit of normal AST based on our home institutions’s guidelines. With the values calculated for APRI from this equation, the four groups in this study were: normal (APRI ≤ 0.5), some liver damage (APRI 0.5 to 0.7), significant fibrosis (APRI 0.7 to 1), and cirrhosis (APRI > 1). [Lin] Statistical analyses were performed to compare demographics, past medical history, and rates of complications between APRI groups. Categorical variables were compared using either Chi-Square Analyses or Fischer’s Exact Tests. Next, multivariate logistic regression analyses were run to determine the relationship between preoperative APRI and postoperative complications. All regressions were adjusted for significant demographics and comorbidities found with bivariate testing. Statistical analyses in this study were performed using SPSS Version 26 (IBM Corp., Armonk, NY, USA). Statistical significance was defined as *p* < 0.05.

## Results

Within the NSQIP database, 275,107 patients were identified who underwent primary THA for osteoarthritis in the years 2007–2020 in the United States. After excluding subjects without preoperative AST and platelet lab values, 129,998 patients remained. Finally, 104,633 total patients were included for analysis in this study, after cases were excluded for missing height/weight, discharge destination, ASA class and baseline functional status (Fig. 1). Of these, 103,678 (99.1%) of subjects were sorted into the normal APRI group, 444 (0.4%) had some liver damage, 256 (0.2%) had significant fibrosis, and 253 (0.2%) had cirrhosis.

Patient sex varied significant between APRI groups, with only 45% males in the normal APRI group compared to 56.3% in the some liver damage group (*p* < 0.001), 59% in the significant fibrosis group (*p* < 0.001), and 54.2% in the cirrhosis group (*p* = 0.004). The distribution of ages across groups also varied, with the normal group have the greatest distribution of individuals above 65 years (normal: 55.1%, some liver damage: 39.9%, significant fibrosis: 34.9%, cirrhosis: 38.7%, all *p* < 0.001). Compared to the normal APRI group (80.4% obese), the some liver damage group had fewer obese patients (75.6% obese) (*p* = 0.031), as did the and the cirrhosis group (70.3) (*p* = 0.002). Moreover, the significant fibrosis group had less functional independence at baseline (6.7% dependent) compared to the normal APRI cohort (2.1% dependent) (*p* < 0.001). Of note, frailty scores did not vary between groups (all *p* > 0.05). Contrarily, ASA class distribution was different between groups, with some liver damage patients (62.8%), significant fibrosis (56.6%) and cirrhosis (67.2%) patients having a significantly greater percentage of subjects with ASA class 3 or higher compared to normal APRI patients (47.6%) (all *p* < 0.05). The abnormal APRI groups also tended to have more smokers (all *p* < 0.001) and bleeding disorders (normal: 2.6% with bleeding disorder, some liver damage: 13.1% with bleeding disorder, significant fibrosis: 13.3% with bleeding disorder, cirrhosis: 16.6% with bleeding disorder, all *p* < 0.001) compared to the normal APRI cohort. Distribution of steroid users, patients with hypertension, congestive heart failure, diabetes and chronic obstructive pulmonary disease did not vary between cohorts (Table 1).

**Table 1:** Patient demographics and comorbidities for patients with preoperative normal APRI, some liver damage, significant fibrosis, and cirrhosis. Bold *p*-values indicate statistical significance with *p* < 0.05

	**Normal (≤ 0.5)**	**Some liver damage (0.5–0.7)**		**Significant fibrosis (0.7–1)**		**Cirrhosis (≥ 1)**	
	**Number (%)**	**Number (%)**	***p*-value**	**Number (%)**	***p*-value**	**Number (%)**	***p*-value**
**Overall**	103,678	444		256		253	
**Sex**			** < 0.001**		** < 0.001**		**0.004**
Female	57,004 (55.0)	194 (43.7)		105 (41.0)		116 (45.8)	
Male	46,674 (45.0)	250 (56.3)		151 (59.0)		137 (54.2)	
**Age**			** < 0.001**		** < 0.001**		** < 0.001**
18–39	2,164 (2.1)	12 (2.7)		7 (2.7)		8 (3.2)	
40–64	44,341 (42.8)	254 (57.3)		159 (62.4)		147 (58.1)	
65–74	34,941 (33.7)	114 (25.7)		56 (22.0)		61 (24.1)	
≥ 75	22,134 (21.4)	63 (14.2)		33 (12.9)		37 (14.6)	
**BMI (kg/m^2)**			**0.031**		0.176		**0.002**
< 18.5	985 (1.0)	8 (1.8)		3 (1.2)		3 (1.2)	
18.5–29.9	19,443 (18.8)	100 (22.5)		60 (23.4)		72 (28.5)	
30–34.9	33,962 (32.8)	123 (27.7)		89 (34.8)		80 (31.6)	
35–39.9	41,564 (40.1)	176 (39.6)		90 (35.2)		84 (33.2)	
≥ 40	7,726 (7.5)	37 (8.3)		14 (5.5)		14 (5.5)	
**Functional Status Prior to Surgery**			0.865		** < 0.001**		0.120
Dependent	2,222 (2.1)	9 (2.0)		17 (6.7)		9 (3.6)	
Independent	101,181 (97.9)	434 (98.0)		237 (93.3)		243 (96.4)	
**Frailty**			0.923		0.457		0.813
< 2	102,223 (98.6)	438 (98.6)		251 (98.0)		249 (98.4)	
≥ 2	1,457 (1.4)	6 (1.4)		5 (2.0)		4 (1.6)	
**ASA classification**			** < 0.001**		**0.004**		** < 0.001**
≤ 2	54,372 (52.4)	165 (37.2)		111 (43.4)		83 (32.8)	
≥ 3	49,308 (47.6)	279 (62.8)		145 (56.6)		170 (67.2)	
**Smoker**			** < 0.001**		** < 0.001**		** < 0.001**
No	90,397 (87.2)	344 (77.5)		186 (72.7)		190 (75.1)	
Yes	13,283 (12.8)	100 (22.5)		70 (27.3)		63 (24.9)	
**Steroid use**			0.829		0.392		0.564
No	98,784 (95.3)	424 (95.5)		241 (94.1)		243 (96.0)	
Yes	4,896 (4.7)	20 (4.5)		15 (5.9)		10 (4.0)	
**Comorbidities**							
**HTN**	60,670 (58.5)	269 (60.6)	0.385	138 (53.9)	0.144	144 (56.9)	0.606
**CHF**	536 (0.5)	4 (0.9)	0.299	2 (0.8)	0.382	1 (0.4)	1.000
**Diabetes Mellitus**	13,968 (13.5)	75 (16.9)	**0.035**	43 (16.8)	0.120	37 (14.6)	0.592
**COPD**	4,634 (4.5)	25 (5.6)	0.238	12 (4.7)	0.879	14 (5.5)	0.363
**Bleeding Disorder**	2,701 (2.6)	58 (13.1)	** < 0.001**	34 (13.3)	** < 0.001**	42 (16.6)	** < 0.001**

*APRI* AST to Platelet Ratio Index, *BMI* body mass index, *ASA* American Society of Anesthesiologists, *CHF* congestive heart failure, *COPD* chronic obstructive pulmonary disease

Regarding complications, major and minor complications and minor complications, were more prevalent in patients with some liver damage (12.8% major complications, 16.9% minor complications), significant fibrosis (14.1% major complications, 17.6% minor complications), and cirrhosis patients (12.6% major complications, 17% minor complications) compared to normal APRI subjects (5% major complications, 8.2% minor complications) (all *p* < 0.001). Rates of pneumonia, bleeding transfusions, readmission, reoperation, non-home discharge and mortality also increased in all abnormal APRI cohorts compared to the normal APRI group (all *p* < 0.05). Pneumonia was more prevalent in some liver damage (1.4%) (*p* = 0.008) and significant fibrosis (1.6%) (*p* = 0.017) patients than in normal APRI patients (0.4%). Similarly, reintubation and failure to wean off the vent were more common in some liver disease and cirrhosis patients compared to the normal APRI group, while the significant fibrosis group was at increased risk for urinary tract infection, superficial incisional surgical site infection and organ site infection (all *p* < 0.05). Finally, periprosthetic fracture was more common in patients with some liver disease (2.5%) (*p* = 0.009) and patients with significant fibrosis (3.5%) (*p* = 0.002) compared to patients with normal APRI (1.1%) (Table 2).

**Table 2:** Bivariate analysis of 30-day postoperative complications in patients with preoperative normal APRI, some liver damage, significant fibrosis, and cirrhosis. Bold *p*-values indicate statistical significance with *p* < 0.05

	**Normal (≤ 0.5)**	**Some liver damage (0.5–0.7)**		**Significant fibrosis (0.7–1)**		**Cirrhosis (≥ 1)**	
	**Number (%)**	**Number (%)**	***p*-value**	**Number (%)**	***p*-value**	**Number (%)**	***p*-value**
**Major Complications**	5,232 (5.0)	57 (12.8)	** < 0.001**	36 (14.1)	** < 0.001**	32 (12.6)	** < 0.001**
**Minor Complications**	8,510 (8.2)	75 (16.9)	** < 0.001**	45 (17.6)	** < 0.001**	43 (17.0)	** < 0.001**
**Sepsis**	280 (0.3)	8 (1.8)	** < 0.001**	3 (1.2)	**0.033**	1 (0.4)	0.496
**Septic Shock**	63 (0.1)	0 (0)	1.000	0 (0)	1.000	2 (0.8)	**0.011**
**Pneumonia**	394 (0.4)	6 (1.4)	**0.008**	4 (1.6)	**0.017**	3 (1.2)	0.074
**Reintubation**	160 (0.2)	4 (0.9)	**0.006**	0 (0)	1.000	4 (1.6)	**0.001**
**Urinary Tract Infection**	962 (0.9)	7 (1.6)	0.139	6 (2.3)	**0.034**	5 (2.0)	0.089
**Stroke**	96 (0.1)	0 (0)	1.000	0 (0)	1.000	1 (0.4)	0.211
**Cardiac Arrest**	91 (0.1)	1 (0.2)	0.323	0 (0)	1.000	0 (0)	1.000
**Myocardial Infarction**	222 (0.2)	2 (0.5)	0.248	0 (0)	1.000	0 (0)	1.000
**Bleeding Transfusions**	6,839 (6.6)	60 (13.5)	** < 0.001**	37 (14.5)	** < 0.001**	40 (15.8)	** < 0.001**
**Deep Vein Thrombosis**	398 (0.4)	2 (0.5)	0.690	3 (1.2)	0.077	1 (0.4)	0.623
**Pulmonary Embolism**	265 (0.3)	1 (0.2)	1.000	0 (0)	1.000	1 (0.4)	0.478
**Failure to wean off ventilator**	63 (0.1)	2 (0.5)	**0.032**	0 (0)	1.000	3 (1.2)	**0.001**
**Deep incisional SSI**	212 (0.2)	4 (0.9)	**0.014**	0 (0)	1.000	1 (0.4)	0.405
**Superficial incisional SSI**	671 (0.6)	6 (1.4)	0.072	5 (2.0)	**0.027**	2 (0.8)	0.681
**Organ/space SSI**	323 (0.3)	3 (0.7)	0.164	6 (2.3)	** < 0.001**	2 (0.8)	0.188
**Wound dehiscence**	0 (0)	0 (0)	1.000	0 (0)	1.000	0 (0)	1.000
**Readmission**	3,904 (3.8)	41 (9.2)	** < 0.001**	28 (10.9)	** < 0.001**	25 (9.9)	** < 0.001**
**Reoperation**	2,121 (2.0)	19 (4.3)	**0.003**	17 (6.6)	** < 0.001**	11 (4.3)	**0.022**
**Home discharge**	84,578 (81.6)	328 (73.9)	** < 0.001**	190 (74.2)	**0.002**	181 (71.5)	** < 0.001**
**Mortality**	254 (0.2)	7 (1.6)	** < 0.001**	4 (1.6)	**0.004**	5 (2.0)	** < 0.001**
**Periprosthetic Fracture**	1,097 (1.1)	11 (2.5)	**0.009**	9 (3.5)	**0.002**	3 (1.2)	0.753
**Dislocation**	488 (0.5)	4 (0.9)	0.160	0 (0)	0.638	2 (0.8)	0.335

*APRI* AST to Platelet Ratio Index, *SSI* surgical space infection

In a multivariate model, patients with some liver damage, significant fibrosis, or cirrhosis had a much higher likelihood of major complication, minor complication, bleeding requiring intraoperative or postoperative transfusion, readmission, and non-home discharge compared to normal APRI patients (all *p* < 0.05). Some liver damage also increased risk for pneumonia (OR: 2.673, 95% CI: [1.174, 6.088], *p* = 0.019), failure to wean off the ventilator (OR: 5.600, 95% CI: [1.339, 23.435], *p* = 0.018) and periprosthetic fracture (OR: 2.254, 95% CI: [1.231, 4.126], *p* = 0.008). Similarly, significant fibrosis increased risk for pneumonia (OR: 3.226, 95% CI: [1.173, 8.867], *p* = 0.023) and periprosthetic fracture (OR: 3.246, 95% CI: [1.656, 6.361], *p* = 0.001). Lastly, cirrhosis was associated with a greater risk of developing septic shock (OR: 7.531, 95% CI: [1.754, 32.346], *p* = 0.007) and experiencing failure of weaning off the ventilator (OR: 12.892, 95% CI: [3.824, 43.458], *p* < 0.001) (Table 3).

**Table 3:** Multivariate analysis of 30-day postoperative complications in patients with preoperative normal APRI, some liver damage, significant fibrosis, and cirrhosis. Bold *p*-values indicate statistical significance with *p* < 0.05

	**Some liver damage (0.5–0.7)**	**Significant fibrosis (0.7–1)**	**Cirrhosis (≥ 1)**
	**OR, 95% CI; *p* value**	**OR, 95% CI; *p* value**	**OR, 95% CI; *p* value**
**Major complications**	2.413, (1.810–3.217); < **0.001**	2.718, (1.883–3.924); < **0.001**	2.314, (1.583–3.384); < **0.001**
**Minor complications**	1.994, (1.538–2.586); < **0.001**	2.329, (1.666–3.255); < **0.001**	1.888, (1.344–2.653); < **0.001**
**Septic Shock**	–	–	7.531, (1.754–32.346); **0.007**
**Pneumonia**	2.673, (1.174–6.088); **0.019**	3.226, (1.173–8.867); **0.023**	2.161, (0.679–6.874); 0.192
**Bleeding transfusions**	1.915, (1.442–2.544); < **0.001**	2.302, (1.603–3.307); < **0.001**	2.125, (1.496–3.019), < **0.001**
**Failure to wean off ventilator**	5.600, (1.339–23.435); **0.018**	–	12.892, (3.824–43.458); < **0.001**
**Readmission**	2.314, (1.666–3.212); < **0.001**	2.738, (1.820–4.120); < **0.001**	2.402, (1.576–3.662); < **0.001**
**Home discharge**	0.600, (0.476–0.756); < **0.001**	0.570, (0.420–0.775); < **0.001**	0.555, (0.412–0.747); < **0.001**
**Periprosthetic Fracture**	2.253, (1.231–4.126); **0.008**	3.246, (1.656–6.361), **0.001**	1.051, (0.335–3.297); 0.932
**Dislocation**	1.601, (0.593–4.326); 0.353	–	1.331, (0.328–5.402); 0.689

*OR* odds ratio, *CI* confidence interval

## Discussion

 Primary THA is an incredibly successful procedure, and, as such, the volume of these cases is rising dramatically in the United States [15]. However, it is important to note that, despite the high level of patient satisfaction and the subsequent ability to regain function, when complications do occur with THA, they can be devastating [16]. In this context, many researchers have sought to identify preoperative risk factors and build evidence-based optimization algorithms around these highlighted problems [17]. The findings of this current, retrospective database study suggest that preoperative APRI can be used to risk stratify patients scheduled to undergo primary THA, while potentially avoiding costly, invasive testing such as a liver biopsy for suspected liver disease, which may cause its own complications and delay care [18]. We found that abnormal APRI, indicative of liver disease ranging from some liver disease to full-blown cirrhosis, is predictive of both major and minor complications in THA patients, as well as unplanned reoperation, non-home discharge, and readmission. Furthermore, specific complications that were seen in abnormal APRI patients included surgical site infections, bleeding requiring transfusion, and periprosthetic fracture. Thus, utilizing preoperative APRI to screen for patients at high-risk for grave postoperative complications following THA will likely improve perioperative care, promote better outcomes, reduce overall hospital costs, and enable physicians to have realistic conversations with the patient about surgical decision-making.

Past research has shown that cirrhosis confers a much greater risk for postoperative complications following THA. For instance, a study by Seol et al. found that there was an overall 30% complication rate and 12% mortality rate within 30 days of THA or total knee arthroplasty in cirrhotic individuals [17, 19]. Similarly, our current study found high rates of major and minor complications in patients with some liver disease, significant fibrosis, and cirrhosis. These rates were much higher than the rate of complications seen in normal APRI patients. It can therefore be concluded that the sequalae of liver disease, even early on in the disease course, were associated with patients’ risks for complications following THA.

Furthermore, in this study, the most common complications were infections, such as pneumonia, urinary tract infection and surgical site infection, along with bleeding requiring transfusion. This is also consistent with the sequalae of liver disease. For instance, cirrhotic patients are said to be at increased risk of infection as they have greater bacterial translocation from the gut, [20] have dysfunction of their immune cells along with complement deficiency and have hyperactivity in cytokine activation after surgery that may be detrimental to healing and immune response [9]. Moreover, as the liver produces platelets and coagulation factors, patients with liver disease have been shown to have the tendency to bleed after THA [21]. Both of these complications can be mitigated by understanding risk in liver disease patients and taking appropriate countermeasures, such as anticipating blood loss and having blood products ready and ensuring close follow-up to check for signs of postoperative infection. In this way, use of APRI can alert physicians and healthcare staff to individuals who may need these additional measures before, during and after primary THA.

Another interesting finding of this current study was an increased risk of periprosthetic fracture in patients with abnormal APRI. It is known that periprosthetic fractures occur more often in patients with poor bone quality, osteolysis or any kind of bone loss [22]. It is also known that cirrhosis may cause hepatic osteodystrophy, a spectrum of bone diseases that includes osteoporosis and/or osteomalacia [23]. Therefore, evidence of the relationship between abnormal APRI and this devastating postoperative THA complication is unsurprising. But evidence of this relationship and the use of APRI in screening high risk patients must be encouraged, as it can help prevent burden to the patient and to the healthcare system as a whole. To put this into context, while average hospital cost for primary THA has been shown to be around $11,104, costs for revision THA are around $14,935 and average loss of revenue to the hospital for the revision procedure is -$401 [24]. In contrast, the blood tests needed to calculate APRI are routinely available at almost all hospitals and are very inexpensive, estimated at just a few dollars each. [WHO Geneva] Thus, screening for liver disease and stratifying patients who are high risk for complication following THA with APRI makes practical sense.

Finally, we also found abnormal APRI to be associated with non-home discharge and readmission to the hospital. This may be related to the high rate of required reintubations, ventilator dependency, and other severe complications, such as stroke and myocardial infarction, that are known to accelerate a patient’s functional and overall health decline [25, 26]. Importantly, non-home discharges have previously been linked with a higher long-term (1 year) mortality rate. Knowing this, APRI should be implemented in a standardized preoperative screening measure and, perhaps, caution should be advised when proceeding with surgery in these patients, as they are at-risk for both short- and long-term morbidity and mortality.

This study is not without its limitations. Although the NSQIP database is a nationally validated database, it may be subject to missing or miscoded data. Additionally, complications and outcomes data were limited to just 30 days within the postoperative period. Moreover, APRI has only been shown to have poor to moderate sensitivity and accuracy for identify hepatitis B-related fibrosis, [27, 28]. Given this, APRI may be less effective for diagnosing fibrosis and cirrhosis in patients with certain comorbid conditions. Further, because a standardized national database was used for this study, the information regarding the etiology of the included patients’ cirrhosis was not available and is therefore a limitation of our study. Lastly, we did not have access to sonographic information to classify patients’ cirrhosis, therefore our classification of liver disease based on APRI may be flawed and labeling of significant fibrosis served as a mere classification not descriptor. Research and developments in the field of hepatology should be followed to understand the true limitations of APRI in the future.

## Conclusions

Medical optimization and risk stratification are vital to surgical planning surrounding primary THA for osteoarthritis. Utilizing APRI to screen for patients who are high risk for major and minor postoperative complications, including infection and bleeding, periprosthetic fracture, non-home discharge, and hospital readmission following THA will likely improve patient outcomes and greatly decrease hospital costs. In conclusion, this cost effective and non-invasive test may help avoid significant morbidity and mortality and allow THA to continue to be one of the most highly successful procedures in the United States.

## Data Availability

The datasets analyzed during the current study are available in the NSQIP repository, https://www.facs.org/quality-programs/data-and-registries/acs-nsqip/.

## Abbreviations

THA: Total hip arthroplasty
APRI: Aspartate aminotransferase to platelet ratio index
NSQIP: American College of Surgeons National Surgical Quality Improvement Program
ASA: American Society of Anesthesiologists
CPT: Current Procedural Terminology
ACS: American College of Surgeons
AST: Aspartate aminotransferase
BMI: Body mass index
